# Improvement of *Escherichia coli* production strains by modification of the phosphoenolpyruvate:sugar phosphotransferase system

**DOI:** 10.1186/1475-2859-4-14

**Published:** 2005-05-16

**Authors:** Guillermo Gosset

**Affiliations:** 1 Departamento de Ingeniería Celular y Biocatálisis, Instituto de Biotecnología, Universidad Nacional Autónoma de México, Apdo. Postal 510-3, Cuernavaca, Mor. 62250, México

## Abstract

The application of metabolic engineering in *Escherichia coli *has resulted in the generation of strains with the capacity to produce metabolites of commercial interest. Biotechnological processes with these engineered strains frequently employ culture media containing glucose as the carbon and energy source. In *E. coli*, the phosphoenolpyruvate:sugar phosphotransferase system (PTS) transports glucose when this sugar is present at concentrations like those used in production fermentations. This protein system is involved in phosphoenolpyruvate-dependent sugar transport, therefore, its activity has an important impact on carbon flux distribution in the phosphoenolpyruvate and pyruvate nodes. Furthermore, PTS has a very important role in carbon catabolite repression. The properties of PTS impose metabolic and regulatory constraints that can hinder strain productivity. For this reason, PTS has been a target for modification with the purpose of strain improvement. In this review, PTS characteristics most relevant to strain performance and the different strategies of PTS modification for strain improvement are discussed. Functional replacement of PTS by alternative phosphoenolpyruvate-independent uptake and phosphorylation activities has resulted in significant improvements in product yield from glucose and productivity for several classes of metabolites. In addition, inactivation of PTS components has been applied successfully as a strategy to abolish carbon catabolite repression, resulting in *E. coli *strains that use more efficiently sugar mixtures, such as those obtained from lignocellulosic hydrolysates.

## Review

Metabolic engineering can be defined as the purposeful modification of cellular activities with the aim of strain improvement [1]. Development of microbial strains for the production of metabolites is based primarily on the application of recombinant DNA technology to alter the properties of the metabolic network by modifying the level of activity or the properties of specific enzymes. These principles have been applied to the generation of a large number of *Escherichia coli *strains, designed for the production of commercially important compounds [2]. Cultures with these engineered strains usually employ media containing glucose. This sugar is nowadays the most utilized raw material in industrial fermentations with *E. coli*, mostly because it is relatively inexpensive and it is the preferred carbon and energy source for this bacterium. As a component of culture media, glucose provides carbon atoms for biomass and product generation. The cell's capacity to uptake and metabolize this carbohydrate has a profound impact on its growth rate and productivity. Thus, it can be expected that modifications to glucose transport systems should have and important impact on the cell's physiology and this, in turn, can either improve or become detrimental in an industrial production context. The purpose of this review is to summarize the characteristics of glucose uptake systems in *E. coli *and discuss examples where their modification in wild type or engineered production strains has resulted in improved performance.

## Glucose transport systems in *Escherichia coli*

**Figure 1:**
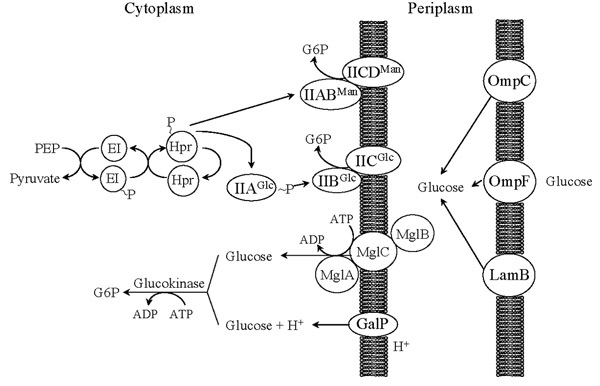
The phosphoenolpyruvate:sugar phosphotransferase system and other glucose transport systems in *Escherichia coli*.

A distinctive feature of *E. coli *and other gram-negative bacteria is the presence of two concentric membranes surrounding its cytoplasm. The space between these two membranes is the periplasm (Fig. 1). The outer and cytoplasmic membranes constitute a hydrophobic barrier to polar compounds. To control the inward and outward flow of molecules across these barriers, the bacterial cell synthesizes proteins that form channels. The outer membrane constitutes the first barrier to the entry of carbohydrates, *E. coli *contains about 10^5 ^channels formed by specialized proteins called porins [3]. The proteins OmpC and OmpF are the most abundant porins present under typical laboratory growth conditions, representing up to 2% of the total cellular protein [4]. Their relative abundance is influenced by various factors such as medium osmolarity, temperature and growth phase [5-7]. It has been shown that these two porins constitute the main entry channels for glucose into the periplasm when this sugar is present at a concentration higher than 0.2 mM [8,9]. In experiments with reconstituted liposomes, the diffusion rate for glucose was found to be about twofold higher through OmpF than through the OmpC channel [10]. Under conditions of glucose limitation, synthesis of the outer membrane glycoporin LamB is induced [9]. This protein permeates several carbohydrates including maltose, maltodextrins and glucose [11]. It has been demonstrated that under external submicromolar concentrations of glucose, LamB contributes about 70% of the total glucose import capacity of the cell [9].

Glucose diffusion by porins through the outer membrane is a passive process. The driving force for glucose internalization into the periplasm is its active transport into the cytoplasm. Due to the presence of active transport systems in the cytoplasmic membrane, it can be assumed that glucose concentration in the periplasm is very low. Once inside the periplasm, glucose can be internalized into the cytoplasm by the phosphoenolpyruvate:sugar phosphotransferase system (PTS). This protein system belongs to the group translocator family of transporters, which are widespread in bacteria and absent in Archaea and eukaryotic organisms [12,13]. PTS participates in the transport and phosphorylation of several sugars. The system is composed of the soluble and non sugar-specific protein components Enzyme I (EI) and the phosphohistidine carrier protein (HPr), encoded by genes *ptsHI *(Fig. 1). These proteins relay a phosphoryl group from PEP to the sugar-specific enzymes IIA and IIB. The last component of this system, IIC (in some cases also IID), is an integral membrane protein permease that recognizes and transports the sugar molecules, which are phosphorylated by component IIB. There are 21 different identified enzyme II complexes encoded in the *E. coli *chromosome, that are involved in the transport of about 20 different carbohydrates [14]. In *E. coli*, the enzyme II complexes II^Glc ^and II^Man ^are involved in glucose import. The glucose-specific II^Glc ^complex is composed of the soluble IIA^Glc ^enzyme and the integral membrane permease IICB^Glc^, encoded by genes *crr *and *ptsG*, respectively. Reported *K*_*m *_and *V*_*max *_values for II^Glc ^with glucose as substrate are in the range of 3–10 μM and 126 μmol min^-1 ^g^-1^, respectively [15,16]. The mannose II^Man ^complex is composed of the IIAB^Man ^homodimer enzyme and the integral membrane permease IICD^Man^. These proteins are encoded in the *manXYZ *operon. In addition to mannose, these proteins can also transport with similar efficiently glucose, fructose, N-acetylglucosamine and glucosamine [17]. Reported *K*_*m *_and *V*_*max *_values for II^Man ^with glucose as substrate are 15 μM and 72 μmol min^-1 ^g^-1^, respectively [16]. In a wild type strain growing with glucose as the carbon source, *ptsG *is induced and the *manXYZ *operon is repressed. Inactivation of the genes encoding the II^Glc ^complex abolishes repression of *manXYZ *and the resulting mutant now transports glucose with the II^Man ^complex, displaying a growth rate corresponding to approximately 84% of that observed in a wild type strain [18].

Glucose can also be actively transported into the cytoplasm by systems that are normally involved in galactose internalization. The genes coding for these transporters and for enzymes involved in galactose metabolism are normally induced by the presence of galactose in the growth medium. However, it has been demonstrated that under growth conditions where external glucose concentration is lower than 1 μM, the genes encoding these systems are maximally induced in the absence of galactose [19,20]. A similar response has been observed in an *E. coli *strain lacking the PTS and growing in a medium with a relatively high glucose concentration (2 g/l) [21]. Analysis of strains growing in glucose-limited conditions revealed that induction of these genes is caused by the intracellular synthesis of galactose that functions as an autoinducer of the system [19]. One of the genes induced under conditions of glucose limitation is *galP*, that encodes the low affinity galactose:H^+ ^symporter GalP. It constitutes an integral membrane protein belonging to the major facilitator superfamily (MFS) of the electrochemical potential-driven class of transporters. The GalP protein can import glucose with a *K*_*m *_of 10.2 μM and a *V*_*max *_of 15.6 μmol min^-1 ^g^-1 ^[22]. The genes in the *mglABC *operon encode an ATP-binding protein, a galactose/glucose periplasmic binding protein and an integral membrane transporter protein, respectively. These proteins constitute the Mgl system, that is also involved in galactose/glucose (methyl galactoside) import. This high affinity porter belongs to the ATP-binding cassette (ABC) superfamily of the primary active class of transporters. The Mgl proteins together with LamB constitute a high-affinity glucose transport system that is induced when this sugar is present at a very low concentration [19]. Glucose internalized by GalP or the Mgl systems must be phosphorylated to enter the Embden-Meyerhof-Parnas (EMP) glycolytic pathway. The enzyme glucokinase, encoded by *glk*, catalyzes the ATP-dependent phosphorylation of glucose in the cytoplasm [23]. Glucokinase activity in *E. coli *is not essential when glucose is abundant and it is transported by PTS. However, in conditions of glucose limitation or in a mutant lacking PTS, inactivation of *glk *severely impairs growth capacity [17].

As it can be observed in figure 1 and Table 1, the energetic costs of importing glucose differ significantly among these systems. Since glucose must be phosphorylated in order to enter the EMP glycolytic pathway, a fair comparison must include transport and phosphorylation reactions. It can be observed that PTS is the most efficient system as it consumes one mol of PEP for each mol of internalized and phosphorylated glucose. The energetic equivalent of one mol of PEP is one mol of ATP, since the conversion of PEP into pyruvate (PYR) by pyruvate kinase would yield a mol of ATP by substrate-level phosphorylation. The high-affinity Mgl-glucokinase system is the most expensive energetically, as it consumes two mol of ATP for every mol of glucose that is internalized and phosphorylated. Finally, GalP and glucokinase transports and phosphorylates glucose at the expense of one mol of H^+ ^that is internalized into the cytoplasm and one mol of ATP.

**Table 1:** Kinetic parameters for glucose transporters and energetic costs for glucose internalization and phosphorylation*.

Transporter	*K*_*m*_	*V*_*max*_	Energetic costs^a^
II^Glc ^complex	3–10 μM	126 μmol min^-1 ^g^-1^	1 PEP
II^Man ^complex	15 μM	72 μmol min^-1 ^g^-1^	1 PEP
GalP	10.2 μM	15.6 μmol min^-1 ^g^-1^	1 H^+ ^+ 1 ATP
MglABC	N.D	N.D.	2 ATP
Glf	4.1 mM	75 μmol min^-1 ^g^-1^	1 ATP

* N.D., not determined.

^a ^The energetic cost equivalent of 1 mol of PEP is 1 mol of ATP.

Laboratory and industrial scale cultures with *E. coli *employ media containing glucose in a wide range of concentrations. Furthermore, feeding strategies are usually implemented to precisely control glucose concentration through different stages in a production process. However, even under these different scenarios, glucose will almost always be present at a concentration that can be transported efficiently by PTS. Thus, it can be assumed that in industrial production fermentations and most laboratory-scale cultures, glucose uptake and phosphorylation will be dependent almost entirely on the II^Glc ^PTS complex. Notwithstanding its efficiency as a transport system, PTS imposes several physiological constraints that can hinder some production processes. In the next sections, the characteristics of PTS that can limit productivity will be discussed and strategies to overcome them will be presented.

## Increasing PEP metabolic availability for the production of shikimate pathway intermediates and amino acids

**Figure 2:**
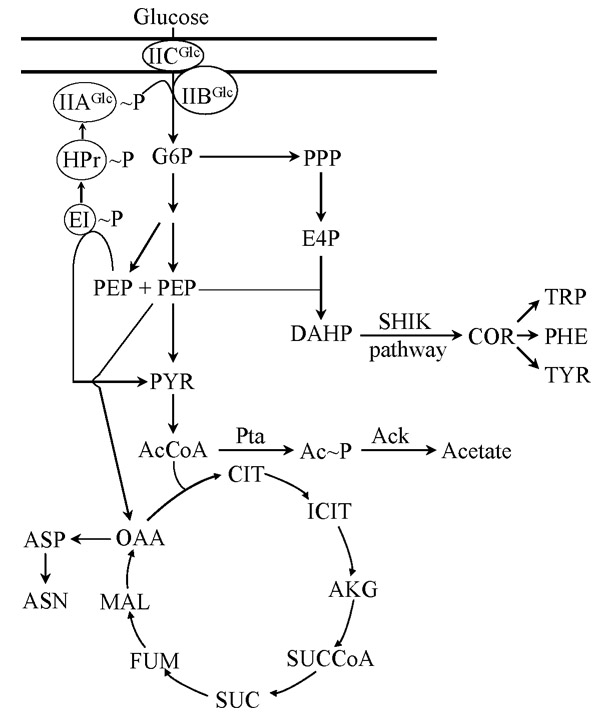
Central pathways related to PTS glucose transport and metabolism.

By coupling glucose internalization to PEP-dependent phosphorylation, the PTS provides a tight linkage between sugar transport and its subsequent metabolism. Fig. 2 shows central metabolism reactions related to PTS activity. It can be observed that PEP is a link between the EMP glycolytic pathway and PTS, together forming a phosphorylation circuit. In addition to its role as a phosphate donor for PTS, PEP participates in several metabolic reactions. It is a precursor in several biosynthetic pathways and also participates directly in energy-generating reactions such as substrate-level phosphorylation of ADP or indirectly as a precursor of acetyl-CoA. The relative carbon flux originating from the PEP node into the different metabolic pathways has been determined experimentally. When *E. coli *grows in minimal medium containing glucose as the carbon source, PTS consumes 50% of available PEP, whereas the reactions catalyzed by PEP carboxylase, pyruvate kinases, UDP-N-acetylglucosamine enolpyruvyl transferase and 3-deoxy-D-*arabino*-heptulosonate 7-phosphate (DAHP) synthase consume approximately 16%, 15%, 16% and 3%, respectively [24-26].

The shikimate (SHIK) or common aromatic pathway is a source of compounds with commercial applications that include the aromatic amino acids tryptophan, tyrosine and phenylalanine. Carbon flux into the common aromatic pathway starts with the condensation of D-erythrose 4-phosphate (E4P) and PEP to yield DAHP, in a reaction catalyzed by the enzyme DAHP synthase (Fig. 2). It has been determined that carbon flux partitioning at the PEP node is the major determinant of yield for aromatic compounds synthesized from glucose in *E. coli *[27-30]. Stoichiometric analyses of the metabolic network involved in aromatics synthesis indicates that the maximum theoretical molar yield from glucose could double in a strain that transports glucose without coupling this process to PEP utilization [31,32]. For this reason, several research groups have explored strategies for developing *E. coli *strains that can uptake glucose without coupling this process to PEP-dependent phosphorylation. Inactivation of the *ptsHI*-*crr *operon is the most common approach for generating PTS^- ^strains. Strains with this genetic modification lack the general PTS proteins involved in the phosphotransfer relay to any of the PTS complexes. Therefore, the resulting PTS^- ^strains will exhibit a very limited capacity to transport and phosphorylate glucose (PTS^-^Glc^- ^phenotype), supported mainly by the GalP, Mgl and glucokinase systems [21]. The severely reduced capacity to transport glucose would be a serious drawback, rendering a PTS^- ^Glc^- ^strain unsuitable for production purposes as it would display very low specific growth rate and productivity. For this reason, different strategies have been investigated for the generation, from PTS^- ^strains, of derivatives that can transport glucose efficiently by a PTS-independent mechanism.

*E. coli *strains lacking a functional PTS but capable of internalizing glucose at a rate similar to that of a PTS^+ ^strain (PTS^- ^Glc^+ ^phenotype), have been selected for their capacity to grow rapidly using glucose as the sole carbon source in a continuous culture [33]. Characterization of these mutants has revealed that glucose is now transported and phosphorylated by an alternative transport system: galactose permease (GalP) and glucokinase (Glk), that uses ATP as phosphate donor instead of PEP [33,26]. These PTS^- ^Glc^+ ^strains have been modified by metabolic engineering to direct carbon flow to the SHIK pathway and they were compared to equally modified PTS^+ ^strains with regard to yield from glucose in the synthesis of DAHP and phenylalanine. These studies showed that the yield from glucose in the synthesis of DAHP and phenylalanine increased by 65% and 57%, respectively, when compared to isogenic PTS^+ ^strains [32-34].

Generation of PTS^- ^Glc^+ ^strains has also been achieved by expressing from a plasmid genes coding for non PTS-dependent glucose transporters. The first example of this approach was the expression of the genes *glf*_*Zm *_and *glk*_*Zm *_encoding, respectively, a glucose facilitator (Glf) and glucokinase from *Zymomonas mobilis *in an *E. coli *strain lacking functional glucose and mannose PTS complexes and glucokinase [35]. The resulting recombinant strain recovered glucose uptake capacity, increasing its specific growth rate from 0.01 to 0.53 h^-1^. The *Z. mobilis *Glf or glucose uniporter belongs to the sugar porter family of the mayor facilitator superfamily. This type of permease does not use energy during transport. Biochemical characterization of Glf revealed that it can transport glucose (*K*_*m *_of 4.1 mM and *V*_*max *_of 75 μmol min^-1^g^-1^) and fructose (*K*_*m *_of 39 mM and *V*_*max *_of 93 μmol min^-1 ^g^-1^) (see Table 1) [36]. More recently, the effects of different expression levels of native *galP *and *glk *on glucose consumption and growth capacity in a PTS^-^Glc^- ^*E. coli *strain were studied [37]. Strain VH32 is an *E. coli *derivative of W3110 having a deletion of the *ptsHI*-*crr *operon that displays a specific growth rate of 0.03 h^-1 ^when using glucose as the only carbon source. When this strain was transformed with a plasmid expressing *glk*, no increase in growth rate was observed. In contrast, expression of *galP *in VH32 increased specific growth rate to 0.55 h^-1 ^and simultaneous expression of both *glk *and *galP *caused VH32 to grow at a specific rate identical to that of the PTS^+ ^parent strain. These results showed that glucose internalization and not phosphorylation is the main limiting factor for rapid growth on glucose for this PTS^- ^Glc^- ^strain. These two approaches for generating PTS^- ^Glc^+ ^strains have been compared with regard to their impact on the yield from glucose in the synthesis of 3-dehydroshikimic acid (DHS) and intermediates from the SHIK pathway in engineered *E. coli *strains [38]. In fed-batch cultures, a strain transporting glucose by PTS synthesized DHS and SHIK pathway intermediates with 33% (mol/mol) yield from glucose. Under the same culture conditions, PTS^- ^strains expressing *glf*_*Zm *_and *glk*_*Zm *_or having glucose transport and phosphorylation dependent on high-level expression of native *galP *and *glk*, synthesized DHS and SHIK pathway intermediates with 41% and 43% (mol/mol) yield from glucose, respectively.

It can be expected that replacement of PTS by an ATP-dependent glucose transport and phosphorylation system should increase the yield from glucose not only for metabolites derived from the SHIK pathway, but also for some compounds having PEP as a precursor. One example is the synthesis of the amino acid asparagine (ASN). This metabolite is synthesized from aspartate (ASP), whose precursor is PEP-derived oxaloacetate (OAA) (Fig. 2). A recent study based on flux balance analysis of gene knockouts or additions in an *E. coli *metabolic model, predicted that replacement of PTS activity by an ATP-dependent glucose transport system should increase asparagine yield by 16.5% [39].

## Reduction of overflow metabolism: acetate production reduction and increasing recombinant protein production

Acetate is one of the fermentation products generated by *E. coli *when it grows using glucose in oxygen-limited conditions. Pyruvate formate-lyase catalyzes the conversion of PYR and coenzyme A (CoA) into acetyl-CoA (AcCoA) and formate. The enzyme phosphotransacetylase (Pta) catalyses an acyl transfer reaction to convert AcCoA and P_i _into acetyl phosphate (Ac~P) and CoA. Finally, acetate kinase (Ack) catalyzes the substrate-level phosphorylation of ADP to yield ATP and acetate. These series of reactions provide the cell with a source of ATP under conditions where aerobic respiration is not possible [40]. However, it is known that *E. coli *can also synthesize a significant amount of acetate under aerobic conditions [41,42]. It has been determined that this condition is the result of the combined high rates of glucose uptake by PTS and glucose catabolism by the EMP pathway that result in a rate of AcCOA synthesis surpassing the capacity of the tricarboxylic acid (TCA) cycle to completely consume this metabolite. Part of the excess AcCOA is diverted into the Ack-Pta pathway to generate acetate [43]. The accumulation of acetate in culture media is an important problem in industrial fermentations since this organic acid inhibits cell growth and recombinant protein production [44]. A common approach to reduce acetate accumulation is the application of glucose feeding strategies to limit glucose availability and the genetic modification of central metabolic pathways directly related to acetate biosynthesis [45,46]. In addition, modification of glucose transport capacity is another successful approach at reducing acetate production rate under aerobic conditions. The metabolically inert glucose analog methyl α-glucoside (α-MG) has been employed as a competitive inhibitor of PTS glucose transport [18]. In this report, the addition of 6.67 g/l of α-MG to *E. coli *cultures growing in complex medium supplemented with 20 g/l of glucose resulted in a 54% reduction in acetate concentration, 39% increase in the specific activity of a recombinant protein and 15% increase in the final biomass concentration, when compared to cultures lacking α-MG. In a different approach, inactivation of *ptsG *was evaluated as a strategy to reduce glucose uptake capacity. In this study, it was determined that cultures of a *ptsG*^- ^strain growing in complex medium supplemented with 15 or 20 g/l of glucose resulted in significant reduction of acetate secretion and more than 50% increase in recombinant protein synthesis when compared to wild type strain cultures [47]. Similar results have been reported with a PTS^- ^Glc^+ ^strain having a deletion of the *ptsHI*-*crr *operon and obtained by selection from a continuous culture [33]. Its characterization when growing in minimal medium cultures with glucose as carbon source revealed that it accumulates 80% less acetate than an isogenic PTS^+ ^strain [48].

**Figure 3:**
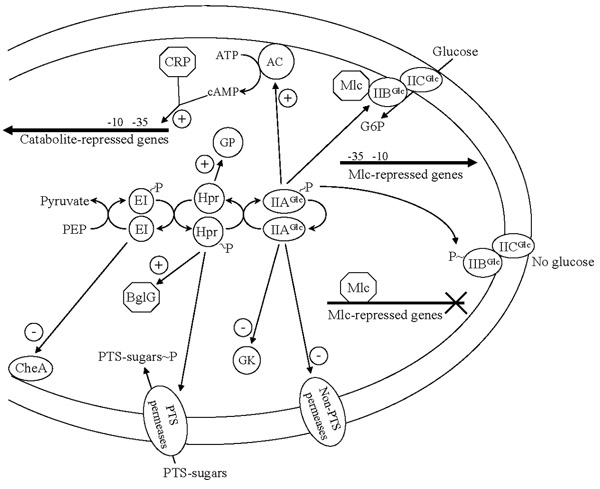
Carbon catabolic repression mechanisms in *Escherichia coli*.

Transcriptional repression of genes encoding PTS components has been reported as a successful method to decrease glucose uptake rate and acetate buildup in wild type *E. coli *strain cultures. The *mlc *gene encodes the regulatory protein Mlc that represses, among others, the *ptsHI *and *ptsG *genes [49] (Fig. 3). Cultures of a recombinant strain having the *mlc *gene expressed from a multicopy plasmid and growing in complex medium with 0.4% glucose, showed a 50% reduction in acetate accumulation and increased capacity to consume this acid when compared to a strain having only a chromosomal copy of *mlc *[50].

## Increasing production of glycolytic and TCA cycle intermediates

The PTS is one of several cellular activities that influences the PEP/PYR ratio and carbon flux distribution originating from these two central metabolic nodes. As the main PEP-consuming activity when *E. coli *grows on glucose or other PTS sugars, it can be expected that modification or elimination of PTS components should have a significant impact on carbon flux distribution in central metabolism. To ascertain some of the effects of PTS inactivation on carbon metabolism, metabolic flux analysis using ^13^C-labeled glucose and NMR spectroscopy has been performed [26]. This study revealed significant carbon flux distribution differences among PTS^+ ^and PTS^- ^strains at the EMP, pentose phosphate pathway (PPP) and TCA cycle. As an example of the results obtained, the relative carbon fluxes into the EMP pathway and the PPP corresponded to 76.6% and 22.3% for the PTS^+ ^strain and 93.1% and 5.3% for the PTS^- ^Glc^+^strain, respectively. Most of the changes observed in metabolic flux distribution in the PTS^- ^Glc^+ ^strain could not have been predicted based on our current knowledge about the properties of the central metabolic network in *E. coli*. Further experimental and theoretical analysis, now in progress by several research groups, will be required to fully understand the connections between PTS and central metabolism.

The effect of PTS replacement by the combined activities of GalP and glucokinase on glycolytic flux to fermentation products has been reported [37]. The high-level expression of *galP *and *glk *in a PTS^- ^Glc^- ^strain restored glucose transport and resulted in a two-fold increase in the specific rate of acetate production when compared to a PTS^+ ^strain. Both of these strains were transformed with a plasmid carrying the *Zymomonas mobilis *genes *pdc*_*Zm *_and *adhB*_*Zm *_encoding pyruvate decarboxylase and alcohol dehydrogenase II, respectively. These enzymes generate a pathway for the synthesis of ethanol from PYR [51]. When these two strains were grown in complex medium supplemented with glucose, a two-fold increase in the specific rate of ethanol production was observed when comparing the PTS^- ^*galP*^+ ^*glk*^+ ^and the PTS^+ ^strains. These results, and those obtained when trying to reduce acetate overflow, show that modulation of *galP *and *glk *expression level in a PTS^- ^Glc^- ^strain allows the possibility of controlling glycolytic flux so that the rate of production of acetate and other fermentation products could be lower of higher than that of a PTS^+ ^strain.

One example of the improvement of a strain for the production of a TCA cycle intermediate is the case of succinate. This is a valuable specialty compound that is employed as a precursor of industrial chemicals [52]. Succinate is currently produced from petroleum derivates as a raw material, but recently, considerable effort has been applied to the development of microbial strains for the biotechnological production of this metabolite. These *E. coli *strains have extensive modifications to central metabolic pathways, resulting in the redirection of carbon flux from the EMP and TCA pathways to enzymes isocitrate lyase (*aceA*) and succinyl-CoA synthetase (*sucC*, *sucD*), both leading to the production of succinate [53]. An additional modification to these strains was the inactivation of *ptsG*. In this study, it was determined that a strain with an inactive *ptsG *displayed a 22.5% increase in final succinate concentration, 16.4% increase in succinate molar yield from glucose and 22% higher specific productivity when compared with an isogenic *ptsG*^+ ^strain. These results have been explained considering that inactivation of *ptsG *caused a lower rate of glucose consumption and acetate production, thus resulting in a more balanced and efficient metabolism [53].

## Elimination of carbon catabolic repression for the simultaneous consumption of sugar mixtures

*E. coli *has the capacity to select, from a mixture of carbon sources, the one that affords the highest growth rate. This response is called carbon catabolite repression (CCR) and it is the result of inhibition of sugar transport capacity, enzyme activities and gene expression by the presence of a rapidly metabolizable carbon source [54]. The IIA^Glc ^protein has a central role in CCR. When glucose is present in the medium, this protein is non-phosphorylated and in this state it binds to various non-PTS permeases, inhibiting uptake of non-PTS sugars (Fig. 3). This form of IIA^Glc ^also binds to the enzyme glycerol kinase (GK), inhibiting its activity [55]. In addition, non-phosphorylated IIB^Glc ^binds the Mlc repressor protein, thus relieving its repression from genes *ptsHI*, *ptsG*, *mlc*, *manXYZ *and *malT *[49]. When glucose is absent from the culture medium, IIA^Glc ^and IIB^Glc ^will be mainly in their phosphorylated state. In this condition, IIA^Glc^~P binds to the enzyme adenylate cyclase (AC) activating its cAMP biosynthetic capacity. Therefore, cAMP concentration increases in the cell, binding to the cAMP receptor protein (CRP) and causing the induction of catabolite-repressed genes [56]. Protein IIB^Glc^~P looses its capacity to bind Mlc, so this protein binds to its target operator sequences, causing repression of genes involved in glucose uptake [49]. Proteins EI and Hpr also have regulatory functions. In its non-phosphorylated state, Hpr activates glycogen phosphorylase (GP), whereas Hpr~P has a similar effect on BglG, a transcriptional activator of the *bgl *operon that encodes proteins involved in β-glucosidic sugars uptake and utilization [57,58]. Non-phosphorylated EI has been shown to bind to the chemotaxis protein CheA, inhibiting its autophosphorylation and thus causing smooth swimming [59].

These data shows that PTS forms part of a regulatory network involved in coordinating cellular processes related to the cell's capacity to find, select, transport and metabolize a large number of carbon sources [12-60]. In addition, it has been demonstrated that protein IIA^Glc ^exerts negative control of expression for the gene encoding the σ^S ^subunit of RNA polymerase [59]. Therefore, it can be expected that alterations to PTS components should have wide-ranging effects on the cell's physiology. Regarding the regulatory functions of PTS, its modification for strain improvement purposes has focused on reducing or eliminating CCR.

Lignocellulose derived from agricultural residues is a potential low-cost feedstock for the production of different types of chemicals by fermentation, among them, fuel ethanol and L-lactic acid [62,63]. Hydrolysis of lignocellulose yields a mixture of sugars containing mainly glucose, arabinose and xylose [64]. Due to CCR, *E. coli *displays sequential sugar consumption when it is grown in media derived from lignocellulose hydrolyzates. Simultaneous consumption of sugars in a mixture would be advantageous in a fermentative production process, as this would eliminate diauxic growth, therefore, reducing operating time and increasing productivity.

Different groups have explored strategies to disrupt CCR by inactivating PTS components. Starting from an *E. coli *strain engineered for increased ethanol production by the introduction of genes *pdc*_*Zm *_and *adhB*_*Zm*_, mutant derivatives were selected on the basis of resistance to the PEP analog fosfomycin [65]. Fosfomycin is used to select for mutants that cannot transport PTS sugars and mutations usually occur in the *ptsI *gene [66]. Some of these mutant strains lost the capacity to ferment PTS sugars, while others retained it. The specific genetic lesions in these strains were not determined. When compared to the parental wild type strain, a mutant strain from the latter class displayed a higher rate of sugars consumption when growing in a medium supplemented with a mixture containing 30 g/l each of glucose, arabinose and xylose. In addition, this mutant produced 20% more ethanol than the wild type when growing in medium containing 120 g/l xylose as sole carbon source [65].

The effect of *ptsG *inactivation on the pattern of sugar mixture utilization and its impact on ethanol production has been determined [67]. In cultures performed with minimal medium containing 2 g/l each of either glucose and arabinose or glucose and xylose, a wild type strain displayed sequential glucose-pentose utilization, whereas the *ptsG *mutant consumed these sugars simultaneously. In both conditions, the *ptsG *mutant consumed the total amount of sugars in about half the time, when compared to the wild type strain. Both strains were transformed with a plasmid bearing the *pdc*_*Zm *_and *adhB*_*Zm *_genes and the transformants were cultured in LB medium supplemented with 4 g/l each of either glucose and xylose. Under these conditions, the ethanol yields of the *ptsG *and wild type strains were 3.5% and 2.9% (w/v), respectively [67]. A similar study was performed with a PTS^- ^Glc^+ ^strain obtained from a PTS^- ^Glc^- ^mutant by a continuous culture selection method [68,33]. When grown in a medium containing 1 g/l each of glucose, arabinose and xylose, the PTS^- ^Glc^+ ^strain consumed the total amount of sugars in the medium 16% faster than an isogenic PTS^+^strain. In addition, acetate produced during growth was completely consumed by the PTS^- ^Glc^+ ^strain, whereas 0.4 g/l remained in the PTS^+ ^strain culture [68].

L-lactic acid is a chemical precursor with many different applications in industry [69]. *E. coli *strains engineered for the production of lactic acid have been developed by inactivation of the genes encoding pyruvate formate lyase and lactate dehydrogenase. The double mutant strain was then transformed with a plasmid carrying a gene encoding the L-specific lactic acid dehydrogenase from *Streptococcus bovis*, thus generating an engineered strain that produces only L-lactate. From this strain, a *ptsG *mutant was generated and compared with other isogenic strains in cultures performed with medium containing 50 g/l each of glucose and xylose. The *ptsG *mutant fermented 75% of the xylose, while for *ptsG*^+ ^strains this value was 18–20%. Furthermore, lactate yield for the *ptsG *and wild type strains were 0.77 (g lactic acid/ g sugar) and 0.48 (g/g), respectively [63].

## Perspectives

As shown by the examples presented here, inactivation of specific PTS components or the total functional replacement of PTS-dependent glucose transport capacity in wild type and engineered strains can result in significant improvements for the production of different classes of compounds. Although some of the strain improvements generated by PTS modification are product-specific, disruption of CCR to improve utilization of sugar mixtures from inexpensive lignocellulosic hydrolysates would be expected to be non product-specific and thus have a positive impact in several of the current biotechnological production processes for bulk and commodity compounds. It would be expected that some of the gains observed by modifying or replacing the glucose PTS components, could also be obtained when the same strategies are applied to the components for other PTS sugars. This is an issue that has not been explored yet, however, it is particularly revelant for production processes based on the use as raw materials of sugar mixtures containing different PTS sugars.

The PTS is widely distributed among Bacteria, therefore, some of the improvements observed in *E. coli *could be translated to other species used in industrial processes like the Gram-positive bacteria *Bacillus subtilis *and *Corynebacterium glutamicum*. Considering the significant differences between Gram-negative and Gram-positive bacteria with regard to central metabolic network architecture, PTS complex composition and CCR regulation, it remains to be determined what would be the consequences of PTS modification in this group of bacteria in an industrial production context.

The PTS is a complex system deeply integrated to the cell's physiology. Much work remains to be done to fully understand the outcome of its modifications. A genomic approach to the study of PTS^- ^strains, including transcriptome, proteome and metabolome analysis, will be fundamental to comprehend the extent of the participation of this system in several of the cell's processes. This knowledge will be valuable to help in the definition of strain design and improvement strategies by metabolic engineering.

## References

[B1] Bailey JE (1991). Toward a science of metabolic engineering. Science.

[B2] Cameron DC, Tong IT (1993). Cellular and metabolic engineering. An overview. Appl Biochem Biotechnol.

[B3] Nikaido H, Nakae T (1979). The outer membrane of Gram-negative bacteria. Adv Microb Physiol.

[B4] Nikaido H, Neidhardt FC (1996). Outer membrane.

[B5] Hall MN, Silhavy TJ (1981). The *ompB *locus and the regulation of the major outer membrane porin proteins of *Escherichia coli *K12. J Mol Biol.

[B6] Lugtenberg B, Peters R, Bernheimer H, Berendsen W (1976). Influence of cultural conditions and mutations on the composition of the outer membrane proteins of *Escherichia coli*. Mol Gen Genet.

[B7] Pratt LA, Silhavy TJ (1996). The response regulator SprE controls the stability of RpoS. Proc Natl Acad Sci U S A.

[B8] Nikaido H, Vaara M (1985). Molecular basis of bacterial outer membrane permeability. Microbiol Rev.

[B9] Death A, Notley L, Ferenci T (1993). Derepression of LamB protein facilitates outer membrane permeation of carbohydrates into *Escherichia coli *under conditions of nutrient stress. J Bacteriol.

[B10] Nikaido H, Rosenberg EY (1983). Porin channels in *Escherichia coli *: studies with liposomes reconstituted from purified proteins. J Bacteriol.

[B11] von Meyenburg K, Nikaido H (1977). Outer membrane of gram-negative bacteria. XVII. Secificity of transport process catalyzed by the lambda-receptor protein in *Escherichia coli*. Biochem Biophys Res Commun.

[B12] Postma PW, Lengeler JW, Jacobson GR, Neidhardt FC (1996). Phosphoenolpyruvate: Carbohydrate phosphotransferase systems. Escherichia coli and Salmonella Cellular and Molecular Biology.

[B13] Saier MH (2000). Vectorial metabolism and the evolution of transport systems. J Bacteriol.

[B14] Tchieu JH, Norris V, Edwards JS, Saier MH (2001). The complete phosphotranferase system in *Escherichia coli*. J Mol Microbiol Biotechnol.

[B15] Misset O, Blaauw M, Postma PW, Robillard GT (1983). Bacterial phosphoenolpyruvate-dependent phosphotransferase system. Mechanism of the transmembrane sugar translocation and phosphorylation. Biochemistry.

[B16] Stock JB, Waygood EB, Meadow ND, Postma PW, Roseman S (1982). Sugar transport by the bacterial phosphotransferase system. The glucose receptors of the *Salmonella typhimurium *phosphotransferase system. J Biol Chem.

[B17] Curtis SJ, Epstein W (1975). Phosphorylation of D-glucose in *Escherichia coli *mutants defective in glucosephosphotransferase, mannosephosphotransferase, and glucokinase. J Bacteriol.

[B18] Chou CH, Bennett GN, San KY (1994). Effect of modulated glucose uptake on high-level recombinant protein production in a dense *Escherichia coli *culture. Biotechnol Prog.

[B19] Death A, Ferenci T (1994). Between feast and famine: endogenous inducer synthesis in the adaptation of *Escherichia coli *to growth with limiting carbohydrates. J Bacteriol.

[B20] Liu X, Ferenci T (1998). Regulation of porin-mediated outer membrane permeability by nutrient limitation in *Escherichia coli*. J Bacteriol.

[B21] Flores N, Flores S, Escalante A, de Anda R, Leal L, Malpica R, Georgellis D, Gosset G, Bolivar F (2005). Adaptation for fast growth on glucose by differential expression of central carbon metabolism and *gal *regulon genes in an *Escherichia coli *strain lacking the phosphoenolpyruvate:carbohydrate phosphotransferase system. Metab Eng.

[B22] McDonald TP, Walmsley AR, Henderson PJF (1997). Asparagine 394 in putative helix 11 of the galactose-H^+ ^symport protein (GalP) from *Escherichia coli *is associated with the internal binding site for cytochalasin B and sugar. J Biol Chem.

[B23] Lunin VV, Li Y, Schrag JD, Iannuzzi P, Cygler M, Matte A (2004). Crystal structures of *Escherichia coli *ATP-dependent glucokinase and its complex with glucose. J Bacteriol.

[B24] Holms WH, Horecker BL, Stadtman ER (1986). The central metabolic pathway of *Escherichia coli*: relationship between flux and control at a branch point, efficiency of conversion to biomass, and excretion of acetate. Current topics in cell regulation.

[B25] Valle F, Muñoz E, Ponce E, Flores N, Bolivar F (1996). Basic and applied aspects of metabolic diversity: the phosphoenolpyruvate node. J Ind Microbiol.

[B26] Flores S, Gosset G, Flores N, de Graff AA, Bolivar F (2002). Analysis of carbon metabolism in *Escherichia coli *strains with an inactive phosphotransferase system by ^13^C labeling and NMR spectroscopy. Metab Eng.

[B27] Förberg C, Eliaeson T, Häggström L (1988). Correlation of theoretical and experimental yields of phenylalanine from non-growing cells of a *rec Escherichia coli *strain. J Biotechnol.

[B28] Varma A, Boesch BW, Palsson BO (1993). Biochemical production capabilities of *Escherichia coli*. Biotechnol Bioeng.

[B29] Patnaik R, Liao JC (1994). Engineering of *Escherichia coli *central metabolism for aromatic production with near theoretical yield. Appl Environ Microbiol.

[B30] Frost JW, Draths KM (1995). Biocatalytic syntheses of aromatics from D-glucose: renewable microbial sources of aromatic compounds. Annu Rev Microbiol.

[B31] Liao JC, Hou SY, Chao YP (1996). Pathway analysis, engineering, and physiological considerations for redirecting central metabolism. Biotechnol Bioeng.

[B32] Báez JL, Bolivar F, Gosset G (2001). Determination of 3-deoxy-D-*arabino*-heptulosonate 7-phosphate productivity and yield from glucose in *Escherichia coli *devoid of the glucose phosphotransferase system. Biotechnol Bioeng.

[B33] Flores N, Yong-Xiao J, Berry A, Bolivar F, Valle F (1996). Pathway engineering for the production of aromatic compounds in *Escherichia coli*. Nat Biotechnol.

[B34] Báez-Viveros JL, Osuna J, Hernandez-Chavez G, Soberon X, Bolivar F, Gosset G (2004). Metabolic engineering and protein directed evolution increase the yield of L-phenylalanine synthesized from glucose in *Escherichia coli*. Biotechnol Bioeng.

[B35] Snoep JL, Arfman N, Yomano LP, Fliege RK, Conway T, Ingram LO (1994). Reconstitution of glucose uptake and phosphorylation in a glucose-negative mutant of *Escherichia coli *by using *Zymomonas mobilis *genes encoding the glucose facilitator protein and glucokinase. J Bacteriol.

[B36] Weisser P, Kramer R, Sahm H, Sprenger GA (1995). Functional expression of the glucose transporter of *Zymomonas mobilis *leads to restoration of glucose and fructose uptake in *Escherichia coli *mutants and provides evidence for its facilitator action. J Bacteriol.

[B37] Hernández-Montalvo V, Martinez A, Hernández-Chávez G, Bolivar F, Valle F, Gosset G (2003). Expression of *galP *and *glk *in a *Escherichia coli *PTS mutant restores glucose transport and increases glycolytic flux to fermentation products. Biotechnol Bioeng.

[B38] Yi J, Draths KM, Li K, Frost JW (2003). Altered glucose transport and shikimate pathway product yields in *E. coli*. Biotechnol Prog.

[B39] Burgard AP, Maranas CD (2001). Probing the performance limits of the *Escherichia coli *metabolic network subject to gene additions or deletions. Biotechnol Bioeng.

[B40] Kessler D, Knappe J, Neidhardt FC (1996). Anaerobic dissimilation of pyruvate. Escherichia coli and Salmonella Cellular and Molecular Biology.

[B41] Han KH, Lim C, Hong J (1992). Acetic acid formation in *Escherichia coli *fermentation. Biotechnol Bioeng.

[B42] Majewski RA, Domach MM (1990). Simple constrained-optimization view of acetate overflow in *E. coli*. Biotechnol Bioeng.

[B43] Delgado J, Liao JC (1997). Inverse flux analysis for reduction of acetate excretion in *Escherichia coli*. Biotechnol Prog.

[B44] Shiloach J, Kaufman J, Guillard AS, Fass R (1996). Effect of glucose supply strategy on acetate accumulation, growth, and recombinant protein production by *Escherichia coli *BL21 (lλDE3) and *Escherichia coli *JM109. Biotechnol Bioeng.

[B45] Konstantinov K, Kishimoto M, Seki T, Yoshida T (1990). A balanced DO-stat and its application to the control of acetic acid excretion by recombinant *Escherichia coli*. Biotechnol Bioeng.

[B46] Yang YT, San KY, Bennett GN (1999). Metabolic flux analysis of *E. coli *deficient in the acetate production pathway and expressing the *B. subtilis *acetolactate synthase. Metab Eng.

[B47] Chou CH, Bennett GN, San KY (1994). Effect of modified glucose uptake using genetic engineering techniques on high-level recombinant protein production in *Escherichia coli *dense cultures. Biotechnol Bioeng.

[B48] Sigüenza R, Flores N, Hernández G, Martínez A, Bolivar F, Valle F (1999). Kinetic characterization in batch and continuous culture of *Escherichia coli *mutants affected in phosphoenolpyruvate metabolism: differences in acetic acid production. W J Microbiol Biotech.

[B49] Plumbridge J (2002). Regulation of gene expression in the PTS in *Escherichia coli *: the role and interactions of Mlc. Curr Opin Microbiol.

[B50] Hosono K, Kakuda H, Ichihara S (1995). Decreasing accumulation of acetate in a rich medium by *Escherichia coli *on introduction of genes on a multicopy plasmid. Biosci Biotechnol Biochem.

[B51] Martinez A, York SW, Yomano LP, Pineda VL, Davis FC, Shelton JC, Ingram LO (1999). Biosynthetic burden and plasmid burden limit expression of chromosomally integrated heterologous genes (*pdc*, *adhB*) in *Escherichia coli*. Biotechnol Prog.

[B52] Zeikus JG, Jain MK, Elankovan P (1999). Biotechnology of succinic acid production and markets for derived industrial products. Appl Microbiol Biotechnol.

[B53] Lin H, Bennett GN, San KY (2005). Metabolic engineering of aerobic succinate production systems in *Escherichia coli *to improve process productivity and achieve the maximum theoretical succinate yield. Metab Eng.

[B54] Saier MH, Ramseier TM, Reizer J, Neidhardt FC (1996). Regulation of carbon utilization. Escherichia coli and Salmonella Cellular and Molecular Biology.

[B55] Novotny MJ, Frederickson WL, Waygood EB, Saier MH (1985). Allosteric regulation of glycerol kinase by enzyme III^glc ^of the phosphotransferase system in *Escherichia coli *and *Salmonella typhimurium*. J Bacteriol.

[B56] Korner H, Sofia HJ, Zumft WG (2003). Phylogeny of the bacterial superfamily of Crp-Fnr transcription regulators: exploiting the metabolic spectrum by controlling alternative gene programs. FEMS Microbiol Rev.

[B57] Seok YJ, Koo BM, Sondej M, Peterkofsky A (2001). Regulation of *E. coli *glycogen phosphorylase activity by HPr. J Mol Microbiol Biotechnol.

[B58] Görke B, Rak B (1999). Catabolite control of *Escherichia coli *regulatory protein BglG activity by antagonistically acting phosphorylations. EMBO J.

[B59] Lux R, Jahreis K, Bettenbrock K, Parkinson JS, Lengeler JW (1995). Coupling the phosphotransferase system and the methyl-accepting chemotaxis protein-dependent chemotaxis signaling pathways of *Escherichia coli*. Proc Natl Acad Sci.

[B60] Saier MH, Reizer J (1994). The bacterial phosphotransferase system: new frontiers 30 years later. Mol Microbiol.

[B61] Ueguchi C, Misonou N, Mizuno T (2001). Negative control of *rpoS *expression by phosphoenolpyruvate: carbohydrate phosphotransferase system in *Escherichia coli*. J Bacteriol.

[B62] Bothast RJ, Nichols NN, Dien BS (1999). Fermentations with new recombinant organisms. Biotechnol Prog.

[B63] Dien BS, Nichols NN, Bothast RJ (2002). Fermentation of sugar mixtures using *Escherichia coli *catabolite repression mutants engineered for production of L-lactic acid. J Ind Microbiol Biotechnol.

[B64] Sheehan J, Himmel M (1999). Enzymes, Energy, and the Environment: A Strategic Perspective on the U.S. Department of Energy's Research and Development Activities for Bioethanol. Biotechnol Prog.

[B65] Lindsay SE, Bothast RJ, Ingram LO (1995). Improved strains of recombinant *Escherichia coli *for ethanol production from sugar mixtures. Appl Microbiol Biotechnol.

[B66] Cordaro JC, Melton T, Stratis JP, Atagun M, Gladding C, Hartman PE, Roseman S (1976). Fosfomycin resistance: selection method for internal and extended deletions of the phosphoenolpyruvate:sugar phosphotransferase genes of *Salmonella typhimurium*. J Bacteriol.

[B67] Nichols NN, Dien BS, Bothast RJ (2001). Use of catabolite repression mutants for fermentation of sugar mixtures to ethanol. Appl Microbiol Biotechnol.

[B68] Hernández-Montalvo V, Valle F, Bolivar F, Gosset G (2001). Characterization of sugar mixtures by an *Escherichia coli *mutant devoid of the phosphotransferase system. Appl Microbiol Biotechnol.

[B69] Jarvis L (2001). Lactic acid outlook up as polylactide nears market. Chem Market Rep.

